# Using Small-Area Estimation to Calculate the Prevalence of Smoking by Subcounty Geographic Areas in King County, Washington, Behavioral Risk Factor Surveillance System, 2009–2013

**DOI:** 10.5888/pcd13.150536

**Published:** 2016-05-05

**Authors:** Lin Song, Laina Mercer, Jon Wakefield, Amy Laurent, David Solet

**Affiliations:** Author Affiliations: Amy Laurent, David Solet, Public Health — Seattle & King County, Seattle, Washington; Laina Mercer, Department of Statistics, University of Washington, Seattle, Washington; Jon Wakefield, Department of Statistics and Department of Biostatistics, University of Washington, Seattle, Washington.

## Abstract

**Introduction:**

King County, Washington, fares well overall in many health indicators. However, county-level data mask disparities among subcounty areas. For disparity-focused assessment, a demand exists for examining health data at subcounty levels such as census tracts and King County health reporting areas (HRAs).

**Methods:**

We added a “nearest intersection” question to the Behavioral Risk Factor Surveillance System (BRFSS) and geocoded the data for subcounty geographic areas, including census tracts. To overcome small sample size at the census tract level, we used hierarchical Bayesian models to obtain smoothed estimates in cigarette smoking rates at the census tract and HRA levels. We also used multiple imputation to adjust for missing values in census tracts.

**Results:**

Direct estimation of adult smoking rates at the census tract level ranged from 0% to 56% with a median of 10%. The 90% confidence interval (CI) half-width for census tract with nonzero rates ranged from 1 percentage point to 37 percentage points with a median of 13 percentage points. The smoothed-multiple–imputation rates ranged from 5% to 28% with a median of 12%. The 90% CI half-width ranged from 4 percentage points to 13 percentage points with a median of 8 percentage points.

**Conclusion:**

The nearest intersection question in the BRFSS provided geocoded data at subcounty levels. The Bayesian model provided estimation with improved precision at the census tract and HRA levels. Multiple imputation can be used to account for missing geographic data. Small-area estimation, which has been used for King County public health programs, has increasingly become a useful tool to meet the demand of presenting data at more granular levels.

## Introduction

King County, Washington, is the 13th largest county in the United States; it had 2.1 million residents in 2014. Although King County fares well in many health indicators compared with other large counties in the United States (1), county-level data mask large disparities among subcounty areas (2). For disparity-focused assessment, a strong demand exists for examining data at the subcounty level, such as census tracts and locally defined areas.

The Behavioral Risk Factor Surveillance System (BRFSS) is the main data source on behavioral risk factors in the United States; it provides state- and county-level estimates and includes zip codes. Although zip codes are useful for subcounty-level analysis, they do not conform to the boundaries of city, county, or other census geographic units such as census tracts. To overcome this limitation, Public Health — Seattle & King County added a “nearest intersection” question to the BRFSS. The intersections are geocoded to define subcounty areas with more granularity and flexibility while protecting respondent confidentiality by not asking for home addresses.

The average BRFSS sample size for King County is approximately 3,300 residents per year. For smaller areas, small sample size becomes an issue. In such situations, small area estimation (SAE) techniques can be used to derive optimal estimates and increase precision (3). In recent years, many SAE studies examined the geographic distribution of health indicators at the county and subcounty levels (3–14). The methods used were synthetic estimation, the head-banging algorithm, multilevel regression, and Bayesian models. The subcounty-level studies showed significant geographic disparities and demonstrated the importance of examining data below the county level.

The objective of this study was to describe how we used geocoded BRFSS data and SAE methods to estimate the prevalence of smoking in King County by census tract and health reporting areas (HRAs). Cigarette smoking was chosen for illustration, but the method may be applied to other indicators.

## Methods

We generated geocoded data in the BRFSS for small-area estimation at the census tract and HRA levels. The modeling method is described here briefly in a less technical way than the modeling method described in the Appendix.

### Generating subcounty data

The BRFSS is a random-digit–dial telephone (cellular and landline) survey of noninstitutionalized adults aged 18 years or older, conducted through collaborations between the Centers for Disease Control and Prevention (CDC) and all 50 US states (15). In Washington State, the Department of Health manages data collection, and the survey is administered in English and Spanish. The department gives organizations an opportunity to add questions beyond the core questions.

Since 1994, information on zip code of the respondent’s residence has been collected by the BRFSS for the King County sample. However, zip code–defined areas are still relatively large, and they do not align well with census tracts or census block-group–based areas such as cities or the King County HRAs. To solve this problem, we added a “nearest intersection” question to the King County BRFSS beginning in 2005 (Box).

**Box. “Nearest Intersection” Question, King County, Washington, Behavioral Risk Factor Surveillance System, 2009–2013**

To help us learn more about environmental factors in your area, we’d like to know what the nearest intersection to your home is. This information will never be released or analyzed individually and will be used to group your responses with others from your neighborhood. Please name the 2 cross-streets of this intersection.

Record first street ___________________________

Record second street _________________________

Don’t know/refused __________________________

### Geocoding

For this analysis of BRFSS data, we examined 5 years of combined data from 2009 through 2013 on 16,283 respondents, excluding 440 respondents with non-King County zip codes or missing data on zip codes. Of the 16,283 respondents, 80% (13,063) answered the nearest intersection question, providing 2 cross street addresses; the literal string variables were first visually corrected and then batch-geocoded by using ArcGIS version 10.1 (ESRI Corp). We manually reviewed each address that did not batch match. We geocoded 12,120 (93%) of the 13,063 residents with nearest intersection data and assigned them to census blocks and census tracts; 74% of all respondents were assigned a geocoded census tract.

#### Data at the census tract level

Of the 399 census tracts in King County, 3 were dropped from the analysis because of their special features (999.99 = boat ramps; 9,999.01 = Puget Sound waterway, and 53.02 = University of Washington campus). During the 5-year period for the 396 remaining census tracts, the sample size ranged from 4 to 108, with a median sample of 28. CDC recommends a minimum sample size of 50 for BRFSS direct estimation (8). Only 12% had a sample size of 50 or larger, which made direct estimation (ie, generating census tract estimates based on the data in each census tract) an unreliable option.

Given that 26% of respondents had missing data on geocodes, we examined imputation methods of census tracts for cases with no geocode but a known zip code. Among the zip codes, the percentage with missing data on census tract varied from 0% to 44%. Multivariate logistic regression showed that among all respondents, missing data on census tract is associated with younger age, nonwhite race/ethnicity, and residing in South King County and East King County. Excluding respondents with missing data on census tracts might mask some of the subcounty disparities at the census-tract level; therefore, we employed a multiple imputation procedure so that all respondents could be included.

#### Data at the health reporting area level

Another common subcounty reporting geographic unit in King County is the HRA, which is defined by census blocks. Forty-eight HRAs encompass individual cities, groups of smaller cities or unincorporated areas, and neighborhoods in larger cities. HRAs are also used to further aggregate King County into 4 regions, the East Region, the South Region, Seattle, and the North Region.

Because the size of HRAs is generally larger than zip code areas, subjects with missing HRAs can be imputed based on zip codes with a relatively lower rate of misclassification than at the census tract level. In addition, routine direct estimation requires fixed HRA designation of the respondents, making the multiple imputation method impractical.

We directly assigned respondents in zip codes with 95% or more of the population in an HRA to that HRA. We randomly assigned remaining subjects with missing HRAs to HRAs based on the distribution of the zip code’s adult population. For example, the adult population in zip code 98001 is 23,324 divided among 3 HRAs: 70.5% in East Federal Way, 20.8% in North Auburn, and 8.7% in Central Federal Way. We randomly assigned respondents in zip code 98001 with missing data on geocode to one of these 3 HRAs by this distribution. After these assignments, the total sample size for HRA-level analysis was 16,176, which accounted for 99% of the total sample.

Sample size for the 5-year combined data ranged from 107 to 642 respondents with a median of 320 per HRA. Although the sample sizes at the HRA level are sufficiently large for direct estimation, SAE models can be used for HRA-level analysis for single-year estimation, for indicators that are limited to subpopulations (eg, mammography screening among women aged 50–74 y), and for other situations where improved precision is desired.

#### BRFSS indicators

Although we applied the SAE models to many BRFSS indicators, for this study, we selected current cigarette smoking for the purpose of demonstration. A current smoker was defined as a respondent who smoked at least 100 cigarettes in his or her entire life and who now smokes every day or some days. Iterative proportional fitting or raking (16) was the method used for generating survey weights, which were based on single years of data and 8 raking margins using King County population estimates.

#### Multiple imputations

Missing census tracts can be imputed on the basis of zip codes. Single imputation methods are fixed or random assignment using certain weights (17,18). However, on average, in King County a zip code contains 7 census tracts. When sample size at the census tract level is relatively small, single imputation methods are subject to high levels of misclassification. In addition, single imputation does not take uncertainty associated with imputation into account and therefore underestimates variance.

Multiple imputation is a method that can reduce differential bias because it does not assign a respondent to a fixed, single census tract. Rather, through an iterative process, respondents with missing data on census tracts are randomly allocated to a census tract within a zip code multiple times. Each multiple allocation is based on the ratio of residential addresses in a census tract to the total number of residential addresses in the entire zip code. We used the ratio provided by the 2011Q1 US Department of Housing and Urban Development zip code-to-census-tract crosswalk table (19). The allocation process was integrated into the SAE model and repeated 100 times. In addition, multiple imputation accounts for the uncertainty of missing data imputation in calculating the standard errors of the estimates (20) (Appendix B).

#### Model used

For the BRFSS indicator at the census tract and HRA levels, we used the hierarchical Bayesian model to obtain smoothed estimates for the 5-year combined period. Our approach summarized the data in each census tract via the asymptotic distribution of the Horvitz–Thompson (21), or direct, estimator of the census tract level proportion. In this way the design is acknowledged in both the estimator and the variance. We defined the area-level data summary as the empirical logistic transform of the direct estimator. This approach constrained the probability to lie in (0,1). This inverse logit transformation allowed us to fit our spatial model but still constrained the prevalence estimates to be between 0 and 1.

We employed 3-stage models; the first stage was given by the asymptotic distribution. The second stage of the model introduced spatial random effects at the census tract or HRA level, which allowed for borrowing information between areas and induced smoothing. The third stage required the selection of hyperparameters (22,23). This approach performed well in an SAE context and was applied to one year of zip code-level BRFSS data (22) and extended for SAE of complex survey data with smoothing in time and space; this approach was applied to estimating child mortality (23). We calculated the sum of log-transformed conditional predictive ordinates to compare various models for the spatial random effects at the second stage. The hierarchical Bayesian model with the highest sum log-conditional predictive ordinates was selected and is described further in Appendix C.

No other covariates were included, because many of the covariates of interest were already accounted for in the raking procedure. The modeling procedures were programmed in the R survey package, version 3.30 (R Foundation for Statistical Computing) (24). Direct estimates were calculated by using the svyglm function of the R survey package (25) from which the design-based variance was extracted. The hierarchical Bayesian space–time models were fitted by using the Integrated Nested Laplace Approximation (INLA) (26) as implemented in the INLA package in R (27). INLA provides a fast alternative to Markov chain Monte Carlo for hierarchical Bayesian models. Appendix D provides details for how the smoothed estimates from each imputed data set were combined to generate the final census tract estimates and credible intervals.

## Results

### Effect of SAE on census tract estimates

Because of the small sample sizes and wide CIs, results from direct estimation for most of the census tracts are unreliable. Of the 396 census tracts, 348 (88%) had a sample size smaller than 50. In addition, 38 census tracts had no respondents self-identifying as a current smoker.

Missing census tract data not only reduced sample size but also could have resulted in biased estimates. Of the 16,283 respondents in the total sample, 4,163 (26%) had missing data on census tracts. Zip codes with 25 or more respondents accounted for 99% of the total sample. Among these zip codes, the percentage with missing census tracts ranged from 0% to 44% with a median of 27%.

For the census tract-level analysis, we compared the rates and half-width of the 90% confidence or credible intervals (CI-hw) estimated by using 3 methods: A) direct, B) smoothed (SAE estimation of cases with geocoded census tract), and C) smoothed plus multiple imputation (SAE estimation based on all cases using multiple imputation for missing census tracts). Method A generates an unreliable and biased estimate, method B is subject to missing data bias, and method C attempts to correct for both the small sample size and missing data problems. We considered 90% to be a programmatically reasonable level of certainty for census tract smoking rates, but the same statistical methods could be used to create more or less conservative intervals, such as 80% or 95%, respectively. Appendix D describes how to create such intervals.

Method A produced rates from 0% to 56% with a median of 10%. The corresponding CI-half-width for census tract with nonzero rates ranged from 1 percentage point to 37 percentage points with a median of 13 percentage points. Rates based on method B ranged from 4% to 26% with a median of 11%, and their corresponding CI-half-width ranged from 3 percentage points to 12 percentage points with a median of 7 percentage points. Method C rates ranged from 5 percentage points to 28 percentage points with a median of 12 percentage points, and the CI-half-width ranged from 4 percentage points to 13 percentage points with a median of 8 percentage points (Table 1). Appendix E presents scatter plots that compare direct estimates with smoothed estimates.

**Table 1:** Summary Statistics for Census Tract-Level Analysis, Prevalence of Smoking by Subcounty Geographies in King County, Washington, BRFSS, 2009–2013

Method	Prevalence,%			Half Width of 90% CI, Percentage Point		
	Minimum	Maximum	Median	Minimum	Maximum	Median
A. Direct	0	56	10	1	37	13
B. Smoothed	4	26	11	3	12	7
C. Smoothed + multiple imputation	5	28	12	4	13	8

Abbreviations: BRFSS, Behavioral Risk Factor Surveillance System; CI, confidence interval.

**Figure 1:**
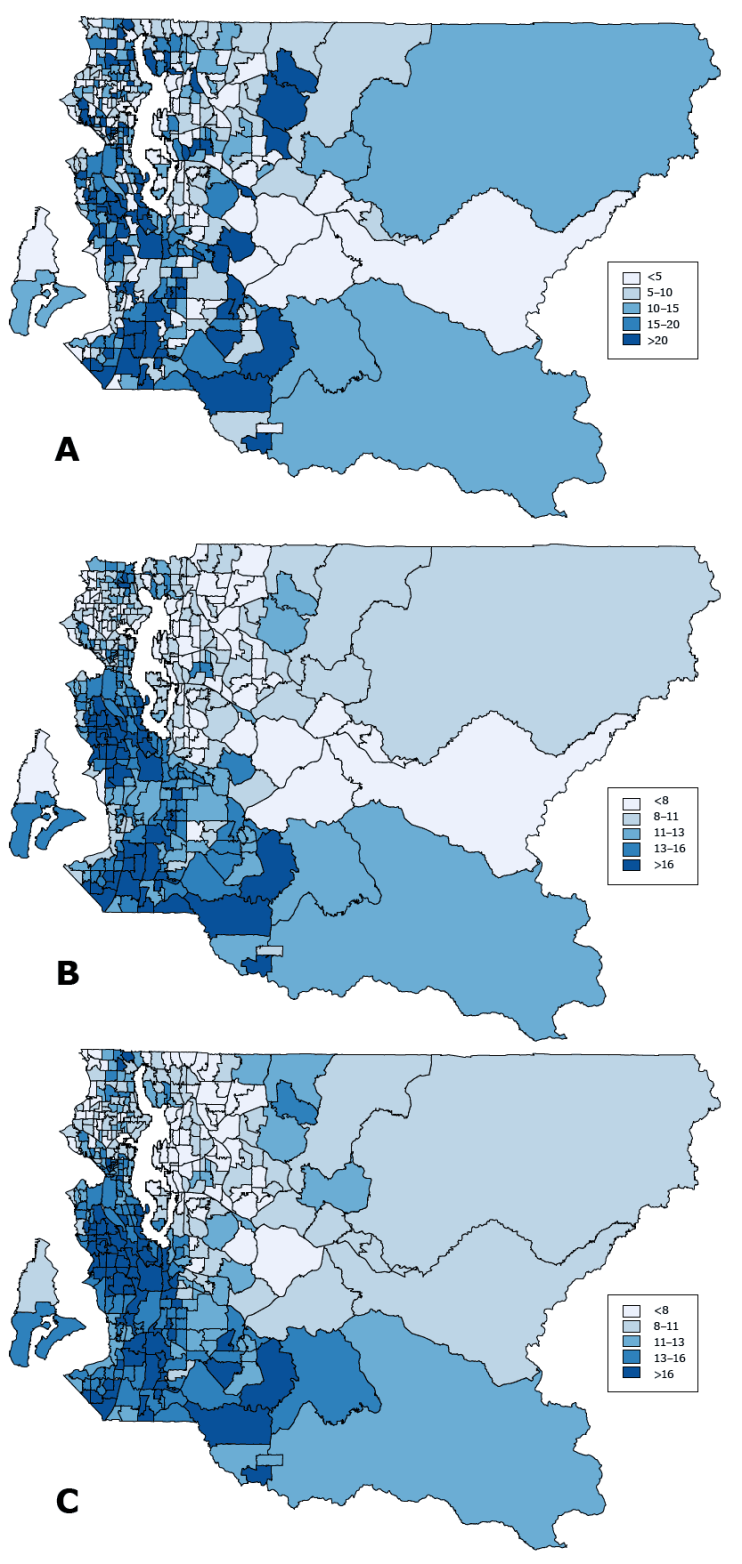
Current smoking prevalence by census tract among King County adults. Maps illustrate 3 methods for estimating smoking prevalence rates by census tract. Map A is based on the direct estimation method. Because sample sizes for many of the census tracts are too small (n <50), prevalence rates estimated by this method are unreliable. Map B shows smoothed estimates derived from our small area estimation model (hierarchical Bayesian model) for respondents with complete information on geocoded census tracts. Respondents with missing data on census tracts were excluded from this analysis. Map C combines the smoothed and the multiple imputation methods to present estimates generated by using both the small area estimation model and multiple imputation to include all respondents. Data are from the King County sample of the Behavioral Risk Factor Surveillance System for 2009 through 2013 combined.

Results varied by geographic estimation methods (Figure 1). By method A, high smoking rates are scattered throughout different regions of the county. By methods B and C, however, high smoking rates are more or less concentrated in south Seattle and South County.

The correlation coefficients are 0.78 between direct and smoothed methods, 0.67 between direct and smoothed–multiple imputation methods, and 0.92 between smoothed and smoothed–multiple imputation methods.

### Effect of small area estimation on health reporting area rates

Among the 48 HRAs in King County, smoking prevalence rates from direct estimation ranged from 5.2% to 30.6% with a median of 13.0%. The 90% confidence interval (CI) half-width ranged from 2.4% to 13.1% with a median of 5.0%. The SAE smoothed rates ranged from 7.2% to 22.8% with a median of 12.5%. The 90% CI-half-width ranged from 2.5% to 7.4% with a median of 3.9%. Table 2 compares smoking rates and 90% uncertainty intervals between the direct estimation method and SAE.

**Table 2:** Current Smoking Prevalence by Health Reporting Area, Direct Method Versus Small Area Estimation (SAE)–Smoothed Method, King County, Washington, BRFSS, 2009–2013

Health Reporting Area	Sample, N	Direct, % (90% CI)	SAE–Smoothed, % (90% CI)
Auburn-North	278	0.22 (0.15–0.31)	0.19 (0.14–0.25)
Auburn-South	181	0.21 (0.14–0.29)	0.19 (0.14–0.25)
Ballard	555	0.09 (0.06–0.14)	0.10 (0.07–0.14)
Beacon/Georgetown/South Park	212	0.14 (0.09–0.21)	0.16 (0.11–0.21)
Bear Creek/Carnation/Duvall	550	0.14 (0.10–0.20)	0.12 (0.09–0.17)
Bellevue-Central	286	0.12 (0.08–0.19)	0.11 (0.08–0.16)
Bellevue-Northeast	319	0.12 (0.08–0.18)	0.11 (0.08–0.14)
Bellevue-South	261	0.07 (0.05–0.11)	0.08 (0.06–0.12)
Bellevue-West	270	0.07 (0.04–0.12)	0.09 (0.06–0.13)
Black Diamond/Enumclaw/Southeast County	395	0.14 (0.11–0.19)	0.14 (0.11–0.18)
Bothell/Woodinville	256	0.13 (0.08–0.21)	0.12 (0.08–0.18)
Burien	434	0.18 (0.14–0.24)	0.18 (0.14–0.23)
Capitol Hill/East Lake	444	0.13 (0.08–0.18)	0.13 (0.09–0.17)
Central Seattle	352	0.14 (0.10–0.20)	0.15 (0.11–0.19)
Covington/Maple Valley	417	0.14 (0.10–0.19)	0.14 (0.11–0.18)
Delridge	221	0.24 (0.17–0.33)	0.21 (0.16–0.28)
Des Moines/Normandy Park	320	0.16 (0.11–0.23)	0.17 (0.12–0.22)
Downtown	298	0.22 (0.16–0.29)	0.19 (0.15–0.24)
East Federal Way	226	0.17 (0.12–0.24)	0.17 (0.13–0.22)
Fairwood	194	0.09 (0.05–0.15)	0.11 (0.08–0.16)
Fed Way-Central/Military Rd	348	0.17 (0.13–0.22)	0.17 (0.13–0.21)
Fed Way-Dash Point/Woodmont	220	0.15 (0.10–0.22)	0.16 (0.11–0.21)
Fremont/Green Lake	445	0.13 (0.09–0.20)	0.12 (0.09–0.17)
Issaquah	251	0.07 (0.04–0.11)	0.08 (0.06–0.12)
Kenmore/Lake Forest Park	361	0.11 (0.07–0.17)	0.11 (0.08–0.15)
Kent-East	172	0.16 (0.11–0.24)	0.16 (0.11–0.21)
Kent-Southeast	405	0.14 (0.10–0.20)	0.15 (0.11–0.19)
Kent-West	175	0.23 (0.14–0.33)	0.19 (0.14–0.25)
Kirkland	481	0.10 (0.06–0.16)	0.10 (0.07–0.14)
Kirkland North	303	0.10 (0.07–0.15)	0.10 (0.08–0.14)
Mercer Isle/Point Cities	349	0.05 (0.03–0.09)	0.08 (0.05–0.12)
Northeast Seattle	642	0.08 (0.06–0.12)	0.09 (0.07–0.12)
Newcastle/Four Creeks	250	0.08 (0.06–0.12)	0.09 (0.07–0.13)
North Highline	105	0.31 (0.19–0.45)	0.22 (0.16–0.31)
North Seattle	495	0.13 (0.09–0.18)	0.12 (0.09–0.16)
Northwest Seattle	434	0.10 (0.07–0.14)	0.10 (0.08–0.14)
Queen Anne/Magnolia	543	0.12 (0.08–0.17)	0.12 (0.09–0.16)
Redmond	404	0.05 (0.03–0.08)	0.07 (0.05–0.10)
Renton-East	219	0.06 (0.03–0.09)	0.08 (0.06–0.12)
Renton-North	185	0.25 (0.17–0.37)	0.17 (0.12–0.23)
Renton-South	333	0.20 (0.15–0.26)	0.18 (0.14–0.23)
Sammamish	397	0.07 (0.05–0.11)	0.08 (0.06–0.11)
Southeast Seattle	290	0.21 (0.15–0.29)	0.18 (0.14–0.24)
SeaTac/Tukwila	295	0.27 (0.20–0.35)	0.23 (0.18–0.29)
Shoreline	527	0.13 (0.10–0.18)	0.12 (0.10–0.16)
Snoqualmie/North Bend/Skykomish	327	0.11 (0.07–0.15)	0.11 (0.08–0.14)
Vashon Island	163	0.12 (0.07–0.21)	0.12 (0.07–0.21)
West Seattle	556	0.10 (0.07–0.15)	0.13 (0.09–0.17)

Abbreviations: BRFSS, Behavioral Risk Factor Surveillance System; CI, confidence interval.

**Figure 2:**
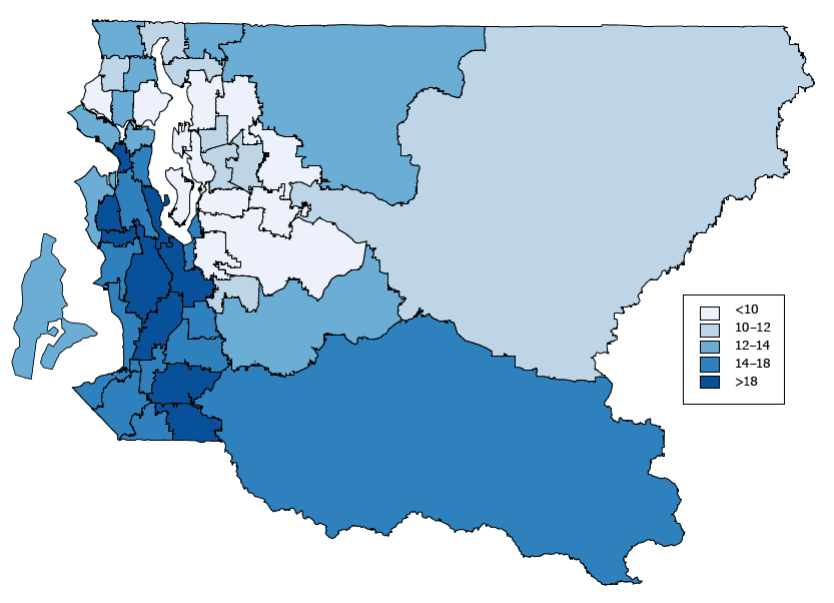
Model-based current smoking prevalence (percentage) among King County adults by King County health reporting areas. The map shows smoothed smoking prevalence rates. Estimates were generated by using a spatial hierarchical Bayesian model. Data are from the King County sample of the Behavioral Risk Factor Surveillance System for 2009 through 2013 combined.

Because the sample sizes at the HRA level are already sufficiently large, the correlation between direct estimates and SAE is high (*r* = 0.96) (Figure 2).

## Discussion

Using the BRFSS data for King County, Washington, we generated hierarchical Bayesian models to estimate the prevalence of current smoking among adults at the level of subcounty geographic areas, including census tracts and HRAs. We defined these more granular geographic areas on the basis of answers to the nearest intersection question we added to the King County BRFSS sample, which provided a convenient method for generating geocoded data while protecting the privacy of survey respondents.

To overcome the problem of small sample size for small areas, we used a spatial Bayesian model to generate smoothed estimation to improve precision. The model also took into account survey weights to adjust for selection bias. Multiple imputation was used to account for missing data in census tracts. The smoothing models did not rely on auxiliary demographic or socioeconomic-status data, making it easier to apply the models to various BRFSS indicators. At the census tract level, the model-generated estimates had better precision than the direct estimates, tightening the median width of the 90% CI from 13 percentage points to 8 percentage points.

A limitation of our study was use of data on nearest intersection as a proxy for actual home address, making misclassification into census tract and HRA a possibility. Although the analysis combined 5 years of data, the precision from the SAE model at the census tract level was still relatively low with somewhat wide 90% CIs. Possible solutions for this limitation could be combining more years of data or aggregating the census tract to even larger areas. Finally, we were unable to identify reliable direct-estimate data to serve as gold standards to validate our SAE census tract results.

The nearest intersection question in the BRFSS can provide geocoded data at subcounty levels; multiple imputation is useful to account for missing census tracts; and SAE is needed to improve precision of estimates. The hierarchical Bayesian model can be useful at the census tract level or larger subcounty geographic levels and can be applied with reasonable precision to the BRFSS indicators for showing place-based disparities. SAE has increasingly become a useful tool to meet the demand of presenting data at more granular levels and is used for our local public health programs in King County.

## Appendix. (Parts A–E)

This appendix is available for download as a Microsoft Word document.
