# Supplementary material for: Using Small-Area Estimation to Calculate the Prevalence of Smoking by Subcounty Geographic Areas in King County, Washington, Behavioral Risk Factor Surveillance System, 2009–2013
Source: Prev Chronic Dis. 2016 May 5;13:E59. doi: 10.5888/pcd13.150536 (PMC4858449; doi:10.5888/pcd13.150536)
Supplement: Supplementary file 1 [file 15_0536_Appendix.docx]

# Appendix A. Notation

- Census tracts are denoted by $i=1,...,I$
- Observations are denoted by $j=1,...,J$ for the $J$
- $y_{j}$ - outcome ($e.g.$ smoking) for observation $j$
- $u_{j}$ - is the census tract for observation $j$
- $w_{j}$ - is the raked sampling weight for observation $j$
- $z_{j}$ - zip code for observation $j$
- $P_{i}$ -True census tract level population prevalence

# Appendix B. Imputation of Census Tract

The Department of Housing and Urban Development (HUD) provides the estimated proportion of a zip code's total residential addresses, which fall within each census tract (CT). For example, if zip code $z$ overlaps with 5 census tracts, we would have $p_{z1},p_{z2},p_{z3},p_{z4},$ and $p_{z5}$ where

$$p_{z1}=\frac{number of residential addresses in zip z and CT1}{total number of residential addresses in zip z}$$

and $\sum_{i=1}^{5} p_{\mathrm{zi}}=1$. We assume that the probability of being from CT $i$ given you are in zip code $z$ is $p_{\mathrm{zi}}$.

For observations with missing census tracts, we impute a census tract based on a multinomial distribution with the HUD probabilities ($p_{\mathrm{zi}}$). For a single imputation each observation $j$ is assigned to census $i$ creating a set of indices $s_{i}$. The imputation is repeated $D$ times, resulting in $d=1,...,D$ complete observation data sets (no missing census tracts).

# Appendix C. Hierarchical Bayesian Model

For each complete data set $d$ our approach is to summarize the data in census tract $i$ via the asymptotic distribution of the estimator of $P_{i}$, which we denote $P_{i}=\sum_{j\in s_{i}} w_{j}y_{j}/\sum_{j\in s_{i}} w_{j}$, the Hajek estimator (1) of $P_{i}$, with corresponding variance estimator var$(P_{i})$. In this way the design is acknowledged in both the estimator and the variance. We define the area-level data summary as $x_{i}=log\left[ P_{i}/(1-P_{i}) \right]$ as the empirical logistic transform of $P_{i}$. This approach constrains the probability to lie in (0,1). The likelihood is then taken as the asymptotic distribution

$$x_{i}|P_{i}\sim N\left( \log\left[ \frac{P_{i}}{1-P_{i}} \right],\frac{\mathrm{var}(P_{i})}{P_{i}^{2}(1-P_{i})^{2}} \right).$$

We employ three-stage models with the first stage given by $x_{i}|P_{i}$ above, which was shown to perform well in a small area estimation context and has been applied to annual zipcode-level BRFSS data (2). When the $P_{i}=0/1$ we have $\mathrm{var}(P_{i})=0$ which is problematic in the first stage of our model. In these cases we use the method of moments based on a beta-binomial model described in the supplementary materials of (3) to provide $P_{i}$ and $\mathrm{var}(P_{i})$ that are adjusted to be non-zero.

At the second stage of the model we introduce the spatial random effects terms, corresponding to the convolution model of (4), and denote the area-specific parameters as

$$\eta_{i}=\mu+\theta_{i}+\phi_{i}$$

where $\mu$ is the overall risk level, $\phi_{i}|\sigma_{\phi}^{2}\sim_{\mathrm{iid}}N(0,\sigma_{\phi}^{2})$ is an independent census tract random effect, and $\theta_{i}$ following an intrinsic conditional autoregressive (ICAR) model (5). The ICAR model is a non-parametric, stochastic smoothing model with

$$\theta_{i}|\theta_{k},k\in ne(i),\sigma_{\theta}^{2}\sim N\left( \overline{\theta}_{i},\frac{\sigma_{\theta}^{2}}{n_{i}} \right),$$

where $ne(i)$ indexes the set of neighbors of area $i$, $n_{i}$ is the number of such neighbors and $\overline{\theta}_{i}=\frac{1}{n_{i}}\Sigma_{k\in ne(i)} \theta_{k}$ is the mean of the neighbors.

For the third stage we use assign Gamma priors on the spatial conditional precision $\sigma_{\theta}^{-2}$ and the $\mathrm{iid}$ precision parameter $\sigma_{\phi}^{-2}$. The rate and shape parameters, 0.5 and 0.008, respectively, were selected such that the 95% range is on the interpretable $\sigma_{\theta}$ and $\sigma_{\phi}$ scale of (0.056,4.04). We use an improper flat prior on $\alpha$.

# Appendix D. Combining Estimates

Our goal is to describe the posterior distribution of $\eta_{i}$ given the observed data. We assume $\mathbf{u}_{o}$ are the set of observed census tracts and $\mathbf{u}_{m}$ are the missing census tracts

$$p(\eta_{i}|\mathbf{y},\mathbf{z},\mathbf{u}_{o})=\int p(\eta,\mathbf{u}_{m}|\mathbf{y},\mathbf{z},\mathbf{u}_{o})p(\mathbf{u}_{m}|\mathbf{y},\mathbf{z},\mathbf{u}_{o})d\mathbf{u}_{m}\approx\frac{1}{D}\sum_{d=1}^{D} p(\eta|\mathbf{y},\mathbf{z},\mathbf{u}_{o},\mathbf{u}_{m}^{(d)})$$

where $\mathbf{u}_{m}^{(d)}\sim p(\mathbf{u}_{m}|\mathbf{y},\mathbf{z},\mathbf{u}_{o})$. In our current implementation $u_{j,m}^{(d)}\sim p(u_{j,m}|z_{j})$ is the multinomial distribution based on HUD data, but in principle could involve other covariates.

Given our set of $D$ smoothed estimates of each $\eta_{i}$ we find the mean posterior estimate

$$E(\eta_{i}|\mathbf{y},\mathbf{z},\mathbf{u}_{o})\approx\frac{1}{D}\sum_{d=1}^{D} \eta_{i,d}=\eta_{i}$$

where $\eta_{i,d}=E(\eta_{i}|\mathbf{y},\mathbf{z},\mathbf{u}_{o},\mathbf{u}_{m}^{(d)})$ is the estimated $\eta_{i}$ from the $d$th complete data set.

Similarly we find a variance

$$V(\eta_{i}|\mathbf{y},\mathbf{z},\mathbf{u}_{o})\approx\frac{1}{D}\sum_{d=1}^{D} V_{i,d}+\frac{1}{D-1}\sum_{d=1}^{D} \left( \eta_{i,d}-\eta_{i} \right)^{2}=V_{i}$$

where $V_{i,d}=V(\eta_{i}|\mathbf{y},\mathbf{z},\mathbf{u}_{o},\mathbf{u}_{m}^{(d)})$ (the posterior variance of $\eta_{i}$ based on the $d$th complete dataset). This variance estimate has contributions from within and between the sets of estimates (1).

Finally, estimates for census tract $i$ are derived from expit($\eta_{i}$) and 95% credible intervals are generated using

$$\left[ \mathrm{expit}\left( \eta_{i}-1.96\times\sqrt{V_{i}} \right),expit\left( \eta_{i}+1.96\times\sqrt{V_{i}} \right) \right].$$

# Appendix E. Scatter Plots Comparing Direct Estimates with Smoothed Estimates By Sample Size

| Point Estimates | CI Width |
| --- | --- |
| Direct to Complete Case Smoothed  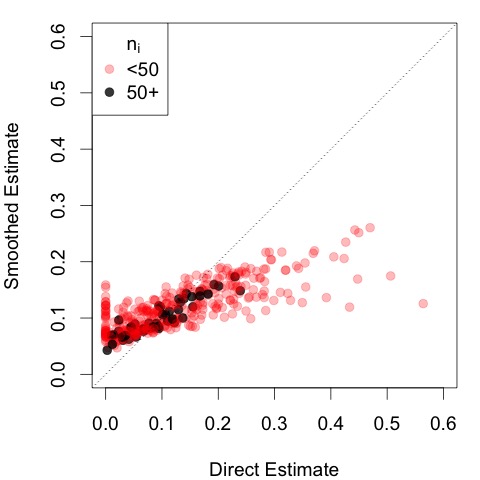 | Direct to Complete Case Smoothed  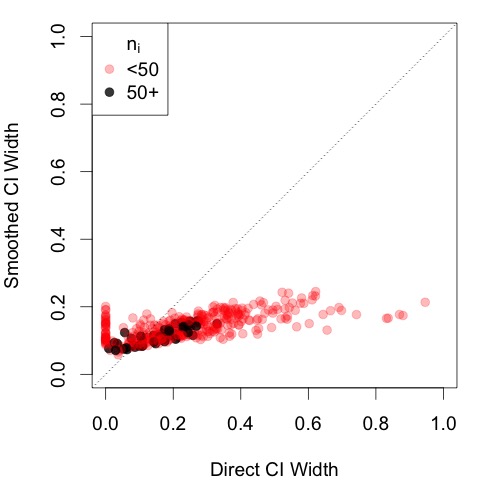 |
| Direct to MI Smoothed  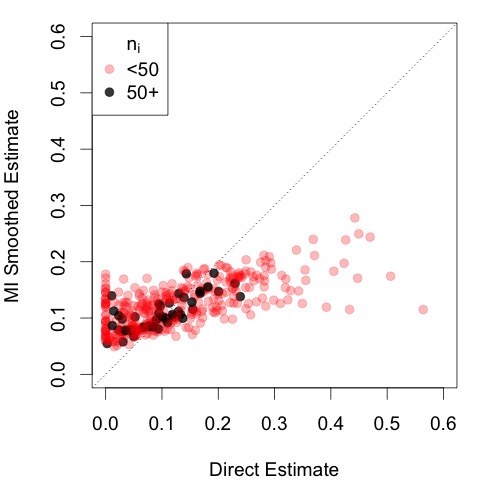 | Direct to MI Smoothed  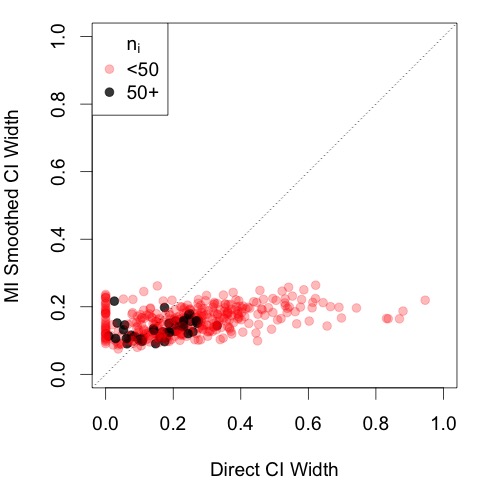 |

**REFERENCES**

1. Hajek, J. (1971). Comments on a paper by D. Basu. In: *Foundations of Statistical*
2. *Inference* (eds: V.P. Godambe and D.A. Sprott). Toronto: Holt, Rinehart and Winston.
3. Mercer LD, Wakefield J, Pantazis A, Lutambi AA, Masanja H, and Clark SJ. “Space-Time Smoothing of Complex Survey Data: Small Area Estimation for Child Mortality”. *Annals of Applied Statistics.* 9(4) *(2015*): 1889-1905.
4. Chen, Cici, Jon Wakefield, and Thomas Lumely. "The use of sampling weights in Bayesian hierarchical models for small area estimation." *Spatial and spatio-temporal epidemiology* 11 (2014): 33-43.
5. Besag, Julian, Jeremy York, and Annie Mollié. "Bayesian image restoration, with two applications in spatial statistics." *Annals of the institute of statistical mathematics* 43.1 (1991): 1-20.
6. Rue, Havard, and Leonhard Held. *Gaussian Markov random fields: theory and applications*. CRC Press, 2005.
